# Elementary Chemistry, Theoretical and Practical

**Published:** 1850-12

**Authors:** 


*Elementary Chemistry, Theoretical and Practical.* By GEO. FOWNES, F. R. S., &c. *Edited with Additions, by* ROBERT BRIDGES, M. D., Professor of Chemistry in the Philadelphia College of Pharmacy, &c. *Third American, from a late London edition, with numerous wood engravings.* Philadelphia, Lea & Blanchard, 1850.

A new edition of the above work, needs no commendation at our hands. It has already taken an elevated stand amongst the text books on this subject. Owing to the death of the author in Jan. 1849, almost the last hours of whose life were expended upon it, the English press was corrected by Dr. H. Bence Jones, while the American edition has been fully brought up to the day by the labors of the Editor, Dr. Bridges, who has added whatever of novelty has since appeared.

